# Cell-based Assay for Recruitment of DDR1 to Collagen-coated Beads

**DOI:** 10.21769/BioProtoc.3339

**Published:** 2019-08-20

**Authors:** Victoria Juskaite, Birgit Leitinger

**Affiliations:** 1 National Heart and Lung Institute, Imperial College London, London, SW7 2AZ, UK

**Keywords:** Discoidin domain receptor, Collagen, Receptor tyrosine kinase, Receptor recruitment, Ligand binding, Phosphorylation, Signaling

## Abstract

The discoidin domain receptors, DDR1 and DDR2, are key signaling receptors for the extracellular matrix protein collagen. The interactions of cells with collagen are difficult to study because of the difficulty to obtain native collagen fibers for *in vitro* studies. Thus, *in vitro* studies often use acid-soluble collagens in the form of single triple helices, which are not representative of the densely packed insoluble collagen fibers found in tissues. In this protocol, we describe a method that allows stimulating DDR1 locally with collagen-coated beads. Latex beads are first coated with acid-soluble collagen, then added to cells expressing DDR1. Recruitment of DDR1 to the beads and collagen-induced DDR1 phosphorylation is visualized by immunofluorescence microscopy on a widefield microscope. In this method, densely packed collagen is presented to cells in an insoluble form. Bead coating is easy to perform, and this method thus presents a straightforward protocol with which to study local recruitment of collagen receptors to insoluble collagen.

## Background

Collagens are insoluble components of the extracellular matrix. Collagens provide structural support for connective tissues, and regulate fundamental cellular processes such as adhesion, proliferation, migration and differentiation (Kadler *et al.*, 2007; Shoulders and Raines, 2009). Collagens mediate these processes through interaction with specific cell surface receptors. Transmembrane collagen receptors include integrins, discoidin domain receptors (DDR1 and DDR2), glycoprotein VI, and leukocyte-associated immunoglobulin-like receptor-1 (Leitinger, 2011). Interaction of these receptors with collagen is often assayed *in vitro* using collagen in solution. However, collagens in solution do not represent the state of collagens found *in vivo*, which may be assembled as dense, insoluble fibers. Cell-based assays based on reconstituted collagen matrices that mimic the physiological organization of collagen in tissues are widely used (*e.g.*, Doyle, 2016; Di Martino *et al.*, 2017). In these assays, cells are added onto (or within) immobilized matrix. While these assays are excellent to study cellular behavior, the kinetics of recruitment of collagen interaction partners are more difficult to study. The method presented here describes local stimulation of cells with collagen presented on latex beads and recruitment of the collagen receptor DDR1 to these beads.

The DDRs are receptor tyrosine kinases (RTKs) that promote a wide range of human diseases including arthritis, organ fibrosis and many forms of cancer (Leitinger, 2014). Little is known about how collagen binding induces DDR kinase activity. Collagen-induced DDR autophosphorylation occurs with unusually slow kinetics (Shrivastava *et al.*, 1997; Vogel *et al.*, 1997). The DDRs are constitutive dimers in the absence of collagen (Noordeen *et al.*, 2006; Mihai *et al.*, 2009; Xu *et al.*, 2014), ruling out the canonical model of ligand-induced RTK dimerization for kinase activation. Conformational changes within a dimer for DDR kinase activation were also ruled out (Xu *et al.*, 2014). We recently reported that collagen induces DDR1 activation through lateral dimer association and phosphorylation between dimers (Juskaite *et al.*, 2017). In the study, we used collagen presented on beads to recruit and phosphorylate DDR1 locally. We demonstrated that when co-expressed with functional DDR1 receptor, ligand-binding defective DDR1 mutants were recruited to the beads and were phosphorylated. These data with collagen-coated beads support the notion that collagen binding to DDR1 leads to lateral association of dimers.

 The method below describes recruitment of DDR1 to collagen-coated beads in Cos-7 cells transiently expressing DDR1. However, it may also be optimized for studying the interaction between collagen and other collagen receptors such as integrins.

## Materials and Reagents

1. Pipette tips
2. 5 ml, 10 ml and 25 ml sterile serological pipettes
3. 1.5 ml Eppendorf tubes (VWR International, catalog number: 211-2130)
4. 24-well plates (Triple Red, catalog number: TCP011024)
5. 13 mm coverslips (VWR International, catalog number: 631-0150)
6. Cling film
7. Aluminum foil
8. Filter
9. Microscope glass slides (Fisher Scientific, catalog number: 12362098)
10. Parafilm (VWR International, catalog number: 291-1213)
11. Cos-7 cells (ATCC), Cells were used up to passage 30
12. DMEM/F12 medium (Life Technologies, catalog number: 21331046)
13. 10,000 U/ml Penicillin and 10,000 μg/ml Streptomycin (Life Technologies, catalog number: 15140122)
14. 10% heat-inactivated fetal bovine serum (FBS, Life Technologies, catalog number: 10500064)
15. OptiMEM (Life Technologies, catalog number: 11058021)
16. Fugene HD (Promega, catalog number: E2311)
17. Bovine serum albumin (BSA, Fisher Scientific, catalog number: 11483823)
18. Collagen I (acid soluble from rat tail; Sigma-Aldrich, catalog number: C-7661)
19. 3 mm latex beads (Sigma-Aldrich, catalog number: LB30-1ML)
20. Paraformaldehyde (Sigma-Aldrich, catalog number:158127)
21. Triton X-100 (Sigma-Aldrich, catalog number: T8787)
22. Prolong Gold Antifade Reagent (Life Technologies, catalog number: P10144)
23. Monoclonal mouse anti-DDR1 IgG1 (7A9) antibody (produced in the lab, Carafoli *et al.*, 2012)
24. Monoclonal rabbit anti-phospho-DDR1 (Tyr513) (Cell Signaling, catalog number: 14531S)
25. Alexa-Fluor 488 goat anti-mouse IgG (Life Technologies, catalog number: A11001)
26. Alexa-Fluor 555 anti-rabbit IgG (Life Technologies, catalog number: A21428)
27. NaCl (Sigma-Aldrich, catalog number: S7653)
28. KH_2_PO_4 _(Sigma-Aldrich, catalog number: P0662)
29. KCl (Sigma-Aldrich, catalog number: P9541)
30. NA_2_HPO_4 _(Sigma-Aldrich, catalog number: S9763)
31. 100x L-Glutamine (Life Technologies, catalog number: 25030081)
32. Tris base (Sigma-Aldrich, catalog number: T1503)
33. NaOH (Sigma-Aldrich, catalog number: S5881)
34. HCl (Sigma-Aldrich, catalog number: 30721-M)
35. 1x PBS, pH 7.4 (see Recipes)
36. Complete DMEM/F12 medium (see Recipes)
37. Serum-free DMEM/F12 medium (see Recipes)
38. Bead coating buffer (see Recipes)
39. 4% paraformaldehyde-freshly prepared (see Recipes)
40. Antibodies solution (see Recipes)

## Equipment

1. Glass beaker
2. Pipettes
3. Pipette controller
4. Stainless steel forceps (Sigma-Aldrich, catalog number: Z168742-1EA)
5. Metal block (Scientific Laboratory Supplies, catalog number: BLO1136)
6. CO_2 _incubator (37 °C, 5% CO_2_; New Brunswick Galaxy 170S)
7. Olympus BX-51 widefield microscope
8. Microcentrifuge (AccuSpin^TM^ MicroR, Fisher Scientific)
9. Plate centrifuge (Eppendorf, model: 5810R)
10. Rotating wheel (Stuart Tube rotator SB2)

## Procedure

- Cell preparation and transfection
*Note: This protocol describes the recruitment of collagen to Cos-7 cells transiently expressing DDR1.*
  1. Seed Cos-7 cells on 13 mm coverslips in 24-well plates at 1 to 3 x 10^4^ cells/ml in 500 μl complete DMEM/F12 medium per well to obtain around 30% confluency the next day.
*Notes:*
    - *Coverslips were sterilized before use by placing coverslips in a glass beaker and heating in a microwave on full power for 5 min. After heating, the beaker was covered with cling film and aluminum foil and stored at room temperature.*
    - *Exact cell number for cell seeding is not important but it is important that cells are less than 50% confluent on the day of transfection.*
  2. After 24 h, transfect cells with relevant DDR1 expression plasmids using Fugene HD according to the manufacturer’s instructions.
*Note: Transfection conditions may need to be optimized but the transfection solution used for cells seeded on 24-well plates consisted of 50 μl/well OptiMEM medium, 0.25 μg/ml DNA and 0.75 μl/well Fugene HD. The transfection solution was incubated for 15 min at room temperature and added to the cells drop-wise. Medium was changed to fresh complete DMEM/F12 1 h before adding transfection mix.*
  3. Change medium to fresh complete DMEM/F12 medium 4 h after transfection and incubate for another 24 h.
  4. Aspirate medium from cells and add 500 μl of serum-free DMEM/F12 medium/well. Incubate for 16 h before stimulation with collagen-coated beads.
- Coating of beads with collagen or control
*Note: Perform bead coating one day before stimulation of cells. We have not tested whether coated beads can be stored for longer times.*
  1. Resuspend the latex beads thoroughly by vortexing and transfer aliquots to 1.5 ml Eppendorf tubes for coating with collagen or control.
*Note: Each well is stimulated with 1 μl starting bead volume. When stimulating multiple wells, scale up the amount of beads for coating. For example, for stimulating 10 wells, aliquot 10 μl beads for coating.*
  2. Wash the bead aliquot by adding 1 ml distilled water and centrifuge at 4,000 rpm (1,236 *× g*) for 1 min. Aspirate supernatant.
*Note: The latex beads are supplied as a suspension composed of polymer particles and water, with small amounts of surfactant, sodium bicarbonate and potassium sulfate. Washing the beads in water is required to remove the other components.*
  3. Resuspend the beads in 1 ml coating buffer and centrifuge at 4,000 rpm (1,236 *× g*) for 1 min. Aspirate supernatant.
  4. Repeat Step B3.
  5. Resuspend beads in 500 μl coating buffer containing 20 μg/ml collagen I or 20 μg/ml BSA control. Incubate overnight on a rotating wheel at 4 °C.
*Note: We have only tested coating up to 20 μl beads. For coating of higher bead volumes, the coating buffer may need to be scaled up.*
  6. After overnight incubation, add 500 μl 1x PBS to beads and centrifuge at 4,000 rpm (1,236 *× g*) for 1 min. Aspirate supernatant to remove unbound collagen.
  7. To wash beads, add 1 ml 1x PBS to beads and centrifuge at 4,000 rpm (1,236 *× g*) for 1 min. Aspirate supernatant.
  8. Add 1 ml 1% (w/v) BSA in 1x PBS to both collagen-coated and control beads. Incubate for 1 h on a rotating wheel at room temperature. Centrifuge at 4,000 rpm (1,236 *× g*) for 1 min. Aspirate supernatant.
  9. Resuspend beads in serum-free DMEM/F12 medium. The beads are now ready to use.
*Note: Each well is stimulated with 1 μl of starting bead volume resuspended in 200 μl serum-free DMEM/F12 medium. For multiple wells, scale up accordingly.*
- Stimulation of cells with collagen-coated beads
  1. Aspirate serum-free medium from Cos-7 cells grown on coverslips in 24-well plates.
  2. Resuspend the prepared collagen-coated or control-coated beads thoroughly by vortexing and add 200 μl resuspended beads per well to the cells.
  3. Centrifuge plate at 1,200 rpm (290 *× g*) for 1 min to settle beads. Incubate at 37 °C for the required amount of time. In our study, we incubated the beads on cells for 10 min to 4 h.
- Cell fixing and staining
*Notes:*
  - *This method explains labeling of surface DDR1 with an antibody against an extracellular epitope, fixing, permeabilization and labeling of phosphorylated DDR1 in Cos-7 cells grown on coverslips after stimulation with collagen-coated beads.*
  - *Other relevant antibodies may be used for labeling different collagen receptors.*
  1. After stimulation, wash coverslips three times each with 500 μl ice-cold 1x PBS.
  2. Take coverslips from each well with forceps and incubate face down with 30 μl primary anti-DDR1 antibody diluted at 2.5 μg/ml in 8% BSA (w/v), 1x PBS on parafilm spread on metal blocks on ice for 1 h to label cell surface DDR1 only. See Figure 1 for the experimental set-up.
  3. Transfer coverslips back to well with cell side facing up and wash coverslips three times each with 500 μl 1x PBS on ice. Aspirate washes.
  4. Fix cells by adding 500 μl 4% paraformaldehyde in 1x PBS in a chemical fume hood for 15 min at room temperature. Discard paraformaldehyde solution.
*Caution: Paraformaldehyde is hazardous, perform fixation in a chemical hood and dispose of paraformaldehyde appropriately.*
*Note: All subsequent steps are performed at room temperature.*
  5. Wash coverslips three times each with 500 μl 1x PBS. Aspirate washes.
  6. Permeabilize cells by adding 500 μl 0.1% (v/v) Triton X-100 in 1x PBS for 5 min. Aspirate buffer.
  7. Block unspecific binding by incubating coverslips with 8% BSA (w/v) in 1x PBS for 1 h.
  8. Take coverslips from each well with forceps and incubate face down on parafilm with 30 μl primary anti-phosho-DDR1 antibody diluted 1:1,000 in 8% BSA (w/v), 1x PBS for 1 h to label phosphorylated DDR1.
  9. Transfer coverslips back to well with cell side facing up and wash coverslips three times each with 500 μl 1x PBS.
  10. Take coverslips from each well with forceps and incubate face down on parafilm with 30 μl appropriate secondary antibodies diluted 1:500 in 8% BSA (w/v), 1x PBS for 30 min. Protect from light.
  11. Transfer coverslips back to well with cell side facing up and wash coverslips three times each with 500 μl 1x PBS.
  12. Mount coverslips on slides with 10 μl Prolong Gold mounting medium.
  13. Airdry coverslips at room temperature overnight. Protect from light. Store at 4 °C or proceed to imaging under a fluorescent microscope.
- Imaging
  Image on a widefield microscope with brightfield capabilities using a 60x oil objective. The beads are imaged using a brightfield channel while total and phosphorylated DDR1 expressed in Cos-7 cells are imaged using appropriate FITC and TRITC fluorescent filters.
  **Figure 1.**
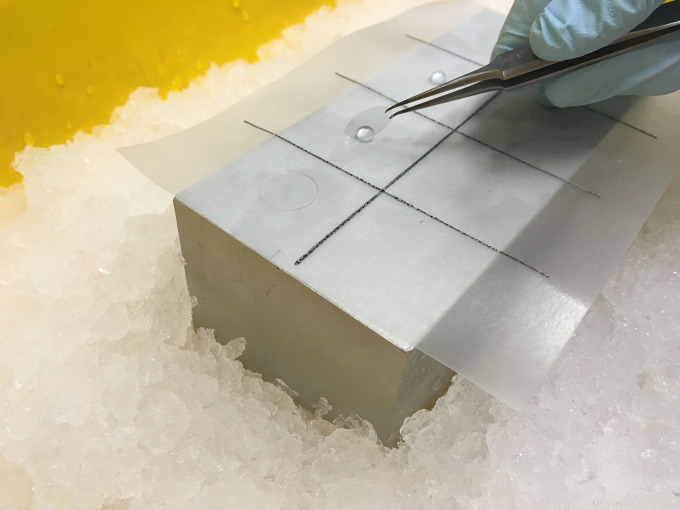
Experimental set-up for incubation of coverslips with primary Ab solution on ice. A metal block is placed onto ice, and a piece of parafilm is placed on top of the metal block. The primary Ab solution is spotted in 30 μl aliquots onto the parafilm, then the coverslips are added with cells face down onto the Ab solution and incubated for 1 h.

## Data analysis

Microscope slides are analyzed by imaging with a widefield microscope using a 60x objective. The images are focused at the interphase of cell and bead contact, and beads, total DDR1 and phosphorylated DDR1 are imaged in brightfield, green and red channels, respectively.

***Representative data***

**Figure 2.**
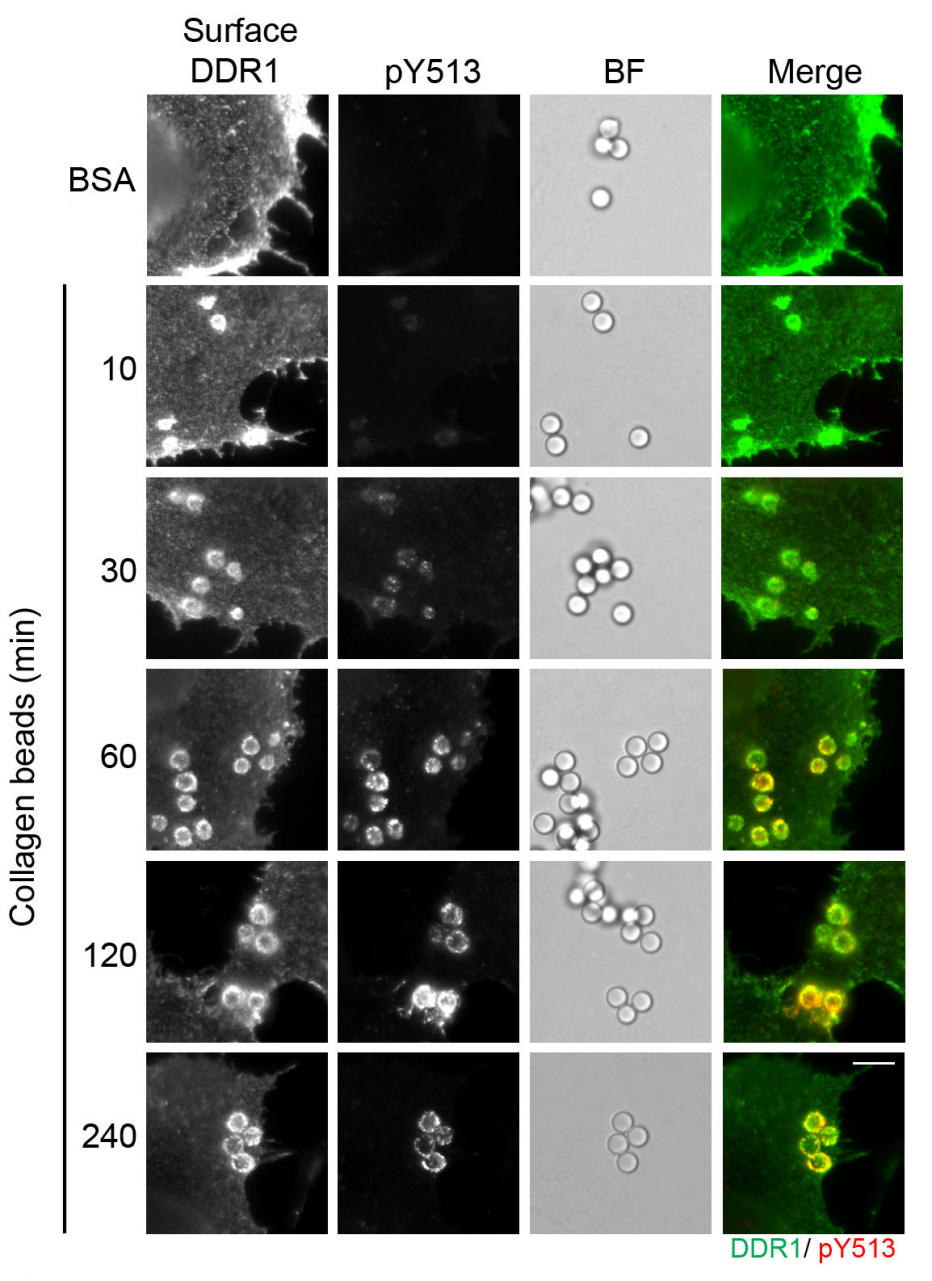
Collagen-coated beads induce local DDR1 phosphorylation on the cell membrane. Wild-type DDR1b was transiently expressed in Cos-7 cells. The cells were incubated with collagen-coated beads for 10 to 240 min at 37 °C, or were incubated with BSA-coated control beads for 240 min, as indicated. The cells were then incubated with a mouse anti-DDR1 Ab, which binds the extracellular discoidin-like domain (Carafoli *et al.*, 2012), on ice to label surface DDR1. Following surface DDR1 labeling, the cells were fixed, permeabilized and incubated with a rabbit anti-phospho-DDR1 (pY-513) Ab, which binds phospho-tyrosine at position 513 within the intracellular juxtamembrane region of DDR1. Afterwards, the cells were incubated with anti-mouse Alexa-Fluor-488 and anti-rabbit Alexa-Fluor-555 secondary Abs, and imaged using an Olympus BX-51 widefield microscope. DDR1 (green) and pY-513 (red) staining are shown in merge image (right panel). BF; Brightfield. Representative images are shown from 3 independent experiments. Scale bar, 10 μm.

In a recent article, we demonstrated recruitment of DDR1 to collagen-coated beads, which led to local DDR1 phosphorylation only (Juskaite *et al.*, 2017). An adapted figure with extra time points is shown below (Figure 2). Data are representative of at least 10 fields of view from 3 independent experiments.

## Recipes

1. 1x PBS, pH 7.4
  0.137 M NaCl
0.018 M KH_2_PO_4_
  0.027 M KCl
0.08 M NA_2_HPO_4_
2. Complete DMEM/F12 medium
  DMEM/F12 medium
  2 mM Glutamine
  10% heat-inactivated fetal bovine serum
  100 U/ml Penicillin and 100 μg/ml streptomycin
3. Serum-free DMEM/F12 medium
  DMEM/F12 medium
  2 mM Glutamine
  100 U/ml Penicillin and 100 μg/ml streptomycin
4. Bead coating buffer
  50 mM Tris (pH 8.5)
  100 mM NaCl
5. 4% paraformaldehyde-freshly prepared (50 ml)
  2 g paraformaldehyde powder
  45 ml distilled water at 65 °C
  20 μl 5 M NaOH
  5ml 10x PBS
  - Incubate paraformaldehyde powder in distilled water at 65 °C to dissolve completely. The powder will not dissolve immediately into solution; increasing the temperature to 65 °C and increasing the pH with NaOH will help to dissolve the powder.
  - Cool at room temperature and add 5 ml of 10x PBS
  - Measure pH and adjust pH to 7.2-7.3 by adding the required amount of 1 N HCl
  - Filter
  - Store unused solution in aliquots at -20 °C
6. Antibody solutions
  Antibodies were diluted in 8% BSA (w/v) in 1x PBS
  Antibody dilutions: 2.5 μg/ml for anti-DDR1, 1:1,000 for anti-phospho-DDR1 and 1:500 (4 μg/ml) for fluorescent secondary antibodies
*Note: For blocking non-specific Ab binding, we used 8% BSA instead of the commonly used 5% BSA concentration, as we found that this condition reduced the background staining of the secondary Abs.*
